# Maternal personality, social support, and changes in depressive, anxiety, and stress symptoms during pregnancy and after delivery: A prospective-longitudinal study

**DOI:** 10.1371/journal.pone.0237609

**Published:** 2020-08-24

**Authors:** Eva Asselmann, Stefanie L. Kunas, Hans-Ulrich Wittchen, Julia Martini

**Affiliations:** 1 Department of Psychology, Faculty of Life Sciences, Humboldt-Universität zu Berlin, Berlin, Germany; 2 Department of Psychiatry and Psychotherapy, Campus Charité Mitte, Charité Universitätsmedizin Berlin, Berlin, Germany; 3 Institute of Clinical Psychology and Psychotherapy, Technische Universität Dresden, Dresden, Germany; 4 Department of Psychiatry and Psychotherapy, Ludwig Maximilians Universität, Munich, Germany; 5 Department of Psychiatry & Psychotherapy, Faculty of Medicine, Carl Gustav Carus University Hospital, Technische Universität Dresden, Dresden, Germany; Sapienza - University of Roma, Italy, ITALY

## Abstract

**Background:**

The role of maternal personality and perceived social support for peripartum changes in psychopathological symptoms remains unresolved.

**Methods:**

In a regional-epidemiological sample of 306 women, depressive, anxiety, and stress symptoms were assessed three times during pregnancy and three times after delivery with the 21-item version of the Depression Anxiety Stress Scale. In pregnancy, the Big Five personality traits and perceived social support were assessed with the short version of the Big Five Inventory and the Social Support Questionnaire.

**Results:**

Multilevel analyses revealed that depressive (*b* = -0.055) and stress (*b* = -0.047) symptoms decreased from early to late pregnancy. After delivery, anxiety symptoms were lower (two months postpartum: *b* = -0.193; four/ 16 months postpartum: *b* = -0.274), but stress symptoms were higher (two months postpartum: *b* = 0.468; four/ 16 months postpartum: *b* = 0.320) than during pregnancy. Across the peripartum period, more conscientious and more extraverted women experienced lower depressive and stress symptoms (*b* = -0.147 to -0.177), and more emotionally stable women experienced lower depressive, anxiety, and stress symptoms (*b* = -0.294 to -0.415). More emotionally stable women more strongly increased in anxiety during pregnancy (*b* = 0.019), and more extraverted women less strongly increased in depression after delivery (*b* = -0.010). Moreover, peripartum depressive, anxiety, and stress symptoms were lower in women with higher perceived social support (*b* = -0.225 to -0.308).

**Conclusions:**

Less emotionally stable, less conscientious, and less extraverted women and women with lower perceived social support seem to be at increased risk for peripartum psychopathological symptoms and might thus particularly profit from targeted prevention.

## Introduction

The transition to motherhood constitutes an important turning point in life. During pregnancy and after delivery, (expectant) mothers experience various physiological changes. Moreover, they need to adjust to their novel family situation, social identity, and role expectations as a mother, which might affect multiple areas of their life such as their partnership [1–3]. After delivery, new mothers need to take care of their newborn on demand and adjust their daily routines and behavior accordingly [4].

### Peripartum changes in depressive, anxiety, and stress symptoms

Dealing with these far-reaching physiological and psychosocial changes might be challenging. Especially in women with specific risk factors (e.g., a previous history of psychopathology), the peripartum period is thus discussed as a vulnerable period for depressive, anxiety, and other peripartum mental disorders [5–9]. At the same time, previous research suggests that unfavorable experiences (e.g., due to psychopathology) during this critical window might have adverse (e.g., epigenetic) effects on both mothers and their offspring, which might at least partially explain the familial transmission of mental disorders [10–12]. Increased psychopathological symptoms during pregnancy and after delivery have been associated with multiple adversities such as pregnancy complications, pre-term birth, altered DNA methylation in mothers and their infants, and impairments of the mother-child-relationship [10–18]. Importantly, such adversities have been linked to an increased risk of developmental problems in the offspring [12, 18–21]. Therefore, individual and psychosocial risk factors for unfavorable changes in peripartum psychopathological symptoms need to be identified. This promises to improve an early recognition of expectant mothers who are particularly likely to experience a symptom escalation and might thus profit from targeted prevention and early intervention [22, 23].

Longitudinal studies often found that depressive and anxiety symptoms (on average) remained stable or even decreased during pregnancy and after delivery [24–31]. Possibly, such favorable changes might relate to a cascade of complex pregnancy-related psychobiological changes that have, for example, been associated with alterations in mood and stress responsivity (e.g., changes in cortisol secretion) [32–36].

Fewer longitudinal studies focused on peripartum changes in stress symptoms [37–41]. Their findings on changes in stress symptoms during pregnancy were mixed [37–40], but point to an increase of stress symptoms from pre- to postpartum [37]. After delivery, new mothers experience a range of far-reaching physiological and psychosocial changes. They need to take care of their child, deal with their novel family situation, identity, and responsibilities, and adjust their daily life accordingly [1–4]. These challenges might lead to an increase of stress symptoms, especially shortly after delivery.

Because of these far-reaching changes it is important (a) to distinguish between different types of symptoms (e.g., depression, anxiety, and stress) and (b) to consider not only continuous, but also discontinuous symptom changes from early pregnancy until several months postpartum [31]. On the one hand, (expectant) mothers might experience a gradual increase or decrease in depressive, anxiety, or stress symptoms during pregnancy and/ or after delivery. On the other hand, they might experience abrupt and transient short-term symptom changes in the first weeks postpartum that attenuate thereafter. For example, new mothers might initially struggle with the physiological (e.g., hormonal) changes after delivery and their novel responsibilities as a mother and thus experience higher depressive, anxiety, and/ or stress symptoms in the early postpartum period. However, they might recover from or adjust to these changes after some time, which might lead to a symptom rebound. Though, also abrupt and enduring long-term symptom changes after delivery are plausible. For example, due to their novel childcare responsibilities, new mothers might experience an immediate increase of distress after delivery, lasting for several months or even years postpartum. Therefore, it is important to take into account continuous and discontinuous short- and long-term changes in depressive, anxiety, and stress symptoms at different junctions across the peripartum period [31].

Many previous studies started in middle or late pregnancy or focused on changes in individual symptoms either before or after delivery. Additional longitudinal studies with multiple waves of assessment are thus needed in order to investigate complex symptom changes over the entire peripartum period, that is, from early pregnancy (as soon as the pregnancy has been confirmed) until more than one year after delivery.

Moreover, the risk to experience peripartum psychopathological symptoms and mental disorders has been shown to vary considerably [42–49] and to be higher in women with elevated psychopathological symptoms in early pregnancy or a previous history of mental disorders or psychiatric treatment [24, 25, 27, 28, 30, 31, 42–45, 48, 50, 51]. However, the role of other factors (e.g., maternal personality and perceived social support) for peripartum changes in depressive, anxiety, and stress symptoms remains unresolved in large parts.

### The role of maternal personality

Personality refers to individual differences in feelings, thoughts, and behavior [52] that can be well described with the Big Five personality traits openness to experience, conscientiousness, extraversion, agreeableness, and emotional stability [53]. Cross-sectional and longitudinal studies evidenced that more emotionally stable women were at lower risk for depressive and anxiety symptoms and disorders during pregnancy and after delivery [54–60]. Some studies also found that higher levels on other Big Five personality traits—especially extraversion [55, 61–63] and conscientiousness [55, 63]—were associated with a lower risk for peripartum depression or anxiety. However, the predictive role of individual Big Five personality traits for changes in depressive, anxiety, and stress symptoms during pregnancy and after delivery remains an open question.

Because emotional stability describes the tendency to experience fewer negative emotions and to be more stress-resistant [64] emotionally stable women might be less susceptible to an escalation of psychopathological symptoms across the peripartum period. More extraverted individuals tend to more actively engage in social interactions and to experience more positive affect [65–67]. Therefore, higher extraversion might relate to higher social support and serve as a buffer against peripartum psychopathological symptoms. Higher conscientiousness has been associated with a higher sense of mastery, purpose, and meaning in life and with more effective self-regulation [64, 68]. For this reason, more conscientious women might be more likely to successfully manage the transition to motherhood.

### The role of perceived social support

Moreover, cross-sectional and longitudinal studies found that women with higher perceived social support were at lower risk for depressive and anxiety symptoms and disorders during pregnancy and after delivery (for review, see [5]). Higher perceived social support also attenuated the association between environmental stressors and symptoms of depression and anxiety during pregnancy [69, 70], and within-person increases in partner and family support predicted subsequent decreases in anxiety and stress symptoms across the peripartum period [71]. Though, additional research is needed to investigate the role of individual differences in perceived social support during pregnancy for more nuanced changes in depressive, anxiety, and stress symptoms during pregnancy and after delivery.

### Aims of the study

In this study, we prospectively followed up a regional-epidemiological sample of 306 (expectant) mothers from Germany from early pregnancy until 16 months postpartum. We modeled continuous and discontinuous short- and long-term changes in depressive, anxiety, and stress symptoms across the peripartum period and examined whether these changes vary by maternal personality and perceived social support in pregnancy.

## Materials and methods

### Procedure

The prospective-longitudinal Maternal Anxiety in Relation to Infant Development (MARI) Study was conducted in 306 expectant mothers, sampled from the community in gynecological outpatient settings in the area of Dresden, Germany (study period: 01/ 2009 until 09/ 2012). Participants were included in the study as soon as their pregnancy had been confirmed and followed up in multiple waves until more than one year postpartum. More specifically, (expectant) mothers (and their infants) completed up to seven assessments: T1 (baseline): week 10 to 12 of gestation; T2: week 22 to 24 of gestation; T3: week 35 to 37 of gestation; T4: 10 days postpartum; T5: two months postpartum; T6: four months postpartum; and T7: 16 months postpartum.

Participants were investigated with standardized diagnostic interviews, questionnaires, and observations. Informed consent was obtained from all participants. The study was approved by the Ethics Committee of the Medical Faculty of the Technische Universität Dresden (No: EK 94042007) and carried out in accordance with the Helsinki Declaration of 1975, as revised in 2013. After consulting the Ethics Committee and due to the sensitive nature of the questions asked in this study and the personal observations of mothers and their infants, participants were assured that all raw data will remain confidential and will not be shared. Therefore, no openly assessable data files are attached. Further information on the data can be obtained from the principal investigator of the MARI study (Prof. Dr. Julia Martini, email: julia-martini@tu-dresden.de), the Ethics Committee of the Medical Faculty of the Technische Universität Dresden (email: ethikkommission@mailbox.tu-dresden.de), and the Institute of Clinical Psychology and Psychotherapy of the Technische Universität Dresden.

**Fig 1:**
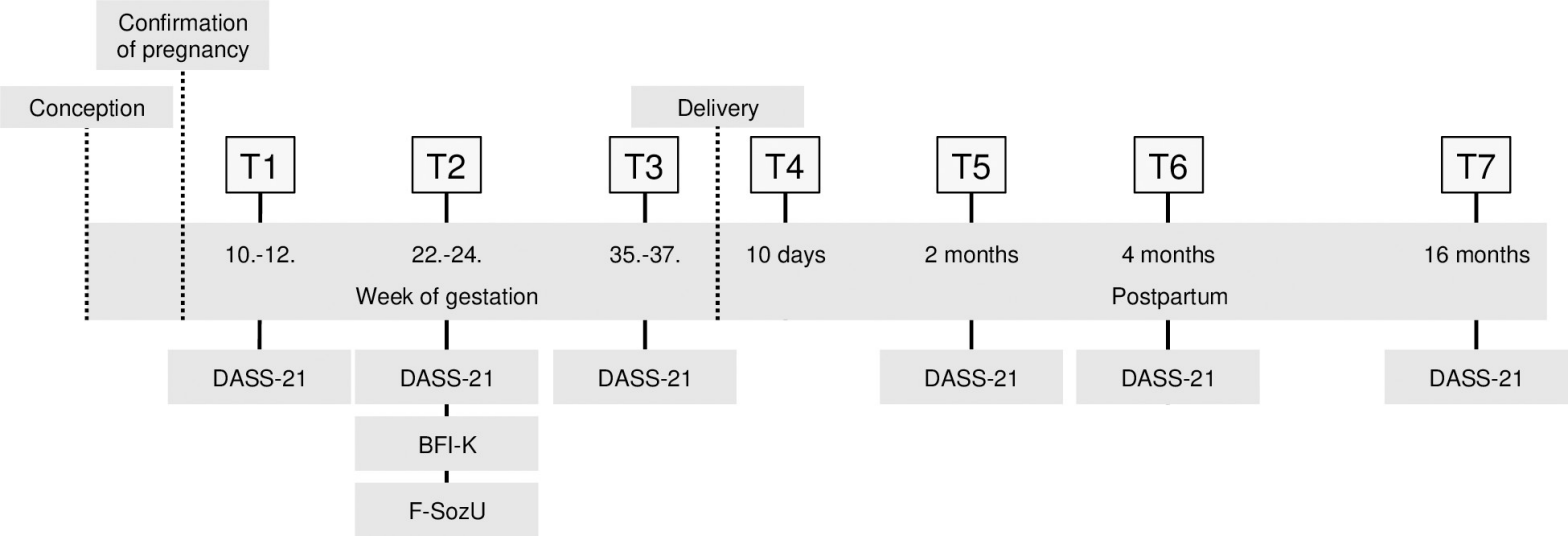
Study design with information when psychopathological symptoms (DASS-21), maternal personality (BFI-K), and perceived social support (F-SozU) were assessed. DASS-21: 21-item short version of the Depression Anxiety Stress Scale; BFI-K: Short version of the Big Five Inventory; F-SozU: Social Support Questionnaire.

More detailed information on the objectives, methods, design, and inclusion/ exclusion criteria as well as a detailed study flow chart have been previously published [6, 72]. The current study design with information on when psychopathological symptoms, maternal personality, and perceived social support were assessed is presented in Fig 1.

### Sample

In total, 533 pregnant women were screened for inclusion and exclusion criteria in gynecological outpatient settings in the area of Dresden (Germany). Fifty women met the exclusion criteria (gestational age>12 weeks: *N* = 8; aged younger than 18 or older than 40 years: *N* = 8; multiple pregnancy: *N* = 2; history of more than three spontaneous abortions, (induced) termination of pregnancy, still birth, or infant impairment: *N* = 2; invasive fertility treatment: *N* = 9; severe physical disease, microsomia, or skeletal malformation: *N* = 6; substance abuse or heroin substitution during the past six months: *N* = 0; severe psychiatric illness: *N* = 2; expectation of leaving the area of Dresden: *N* = 6; insufficient mastery of the German language: *N* = 7). In addition, nine women did not participate due to spontaneous abortions before T1, ten because their partner did not agree, 154 due to a lack of time, and four due to unknown reasons.

Finally, 306 women were enrolled in the study, and 274 women were retained until T7 (retention rate: 89.5%). More specifically, 306 women participated at T1, 293 at T2, 278 at T3, 284 at T4, 281 at T5, 283 at T6, and 267 at T7. Due to a spontaneous abortion or a termination of the pregnancy, the participation of eight women ended after T1. During the study, three women moved away, five women could not be reached anymore by phone, postal, or personal contact, nine women reported a lack of time or interest in a further study participation, and seven women refused to be contacted again for additional follow-up assessments. Some retained women did not participate in single assessments, for example, due to a preterm delivery, sickness, or a lack of time (T3: *N* = 10; T4: *N* = 2; T5: *N* = 5; T6: *N* = 1; T7: *N* = 7). Detailed information on sociodemographic, gynecological, and clinical characteristics of the study sample, including information on systematic drop out from T1 to T7 has been previously presented [6, 72].

### Assessment of depressive, anxiety, and stress symptoms

Depressive, anxiety, and stress symptoms were assessed at each wave (T1, T2, T3, T5, T6, and T7) except for T4 (shortly after delivery) with the German 21-item version of the Depression Anxiety Stress Scale (DASS-21) [73]. The DASS-21 was not applied at T4 because T4 was conducted 10 days postpartum and only included selected assessments to not overstrain participants shortly after the birth of their child.

In line with the tripartite model of depression, anxiety, and stress [74], the DASS-21 assesses depressive, anxiety, and stress symptoms with seven items per symptom subscale, labeled from 0 = ‘seldom/ not at all’ to 3 = ‘mostly/ all the time’. The depression subscale refers to symptoms of anhedonia and inactivity (e.g., ‘I couldn’t seem to experience any positive feelings at all.’). The anxiety subscale covers symptoms of physiological hyperarousal and specific anxieties (e.g., ‘I felt scared without any good reason.’). The stress subscale refers to general distress (e.g., ‘I found it hard to wind down.’). Because the DASS-21 is a short version of the original 42-item DASS [75] the sum scores for each subscale (depression, anxiety, and stress) need to be multiplied by two.

Reliability and validity of the DASS-21 have been shown to be high in both clinical and non-clinical adult populations [76–80]. For example, internal consistencies as well as convergent and discriminant validity of the DASS-21 have been shown to be high, and the three-factor structure has been supported [76–80]. In this study, the internal consistency (Cronbach’s alpha) for depression was α = .74 at T1, α = .68 at T2, α = .73 at T3, α = .77 at T5, α = .80 at T6, and α = .81 at T7. For anxiety, the internal consistency was α = .61 at T1, α = .67 at T2, α = .62 at T3, α = .65 at T5, α = .80 at T6, and α = .72 at T7. For stress, the internal consistency was α = .78 at T1, α = .78 at T2, α = .81 at T3, α = .80 at T5, α = .83 at T6, and α = .84 at T7.

### Assessment of maternal personality

The Big Five personality traits openness, conscientiousness, extraversion, agreeableness, and emotional stability were assessed at T2 using the German short version of the Big Five Inventory (BFI-K) [81]. The BFI-K contains 21 items (five items for openness and four items of each of the other traits), labeled from 1 = ‘strongly disagree’ to 5 = ‘strongly agree’. Example items are ‘I see myself as someone who values artistic, aesthetic experiences.‘ for openness, ‘…does a thorough job.’ for conscientiousness, ‘…is outgoing, sociable.’ for extraversion, ‘…is generally trusting.’ for agreeableness, and ‘…is relaxed, handles stress well.’ for emotional stability.

The reliability of the BFI-K has been shown to be acceptable. The factor structure as well as convergence of self-reports with partner ratings and other inventories have been supported [81]. In this study, the internal consistency (Cronbach’s alpha) was α = .65 for openness, α = .62 for conscientiousness, α = .77 for extraversion, α = .52 for agreeableness, and α = .72 for emotional stability at T2.

### Assessment of perceived social support

Perceived social support was assessed at T2 using the short version (K-14) of the Social Support Questionnaire (F-SozU) Form A [82]. The F-SozU Form A K-14 contains 14 items (labeled 1 = ‘does not apply’, 2 = ‘does rather not apply’, 3 = ‘does partially apply’, 4 = ‘does apply’, and 5 = ‘does exactly apply’). These items refer to practical and emotional support, social integration, satisfaction with social support, and availability of confidants. Example items are ‘I experience a lot of understanding and security from others.’ and ‘I know a very close person whose help I can always count on.’. The total score indicates participants’ overall levels of perceived social support.

The internal consistency (α = .94), test-retest reliability (r = .96 over a period of one week), as well as convergent and discriminant validity of the F-SozU have been shown to be good [82]. In this study, the internal consistency of perceived social support at T2 was high with a Cronbach’s alpha of α = .92. Further information on psychometric properties of the F-SozU has been previously reported [82].

### Assessment of maternal depressive and anxiety disorders

Maternal lifetime depressive and anxiety disorders prior to pregnancy were assessed at T1 with the lifetime version of the Composite International Diagnostic Interview for Women (CIDI-V) [83]. At baseline, 113 women fulfilled criteria for any lifetime depressive disorder prior to pregnancy and 149 women met criteria for any lifetime anxiety disorder prior to pregnancy. Of these women, 65 were affected by both, any lifetime depressive disorder prior to pregnancy and any lifetime anxiety disorder prior to pregnancy. More detailed information hereon has been previously presented [6, 72].

### Statistical analyses

We used Stata 14 [84] for the analyses and applied multilevel analyses with measurement occasions (Level 1) nested within persons (Level 2). Specifically, we simultaneously regressed the standardized score of the respective symptom subscale of the DASS-21 on linear, quadratic, and cubic age as well as four event-related predictors (linear symptom changes during pregnancy, linear symptom changes after delivery, short-term symptom changes after delivery, and long-term symptom changes after delivery). These event-related predictors were used to model different types of continuous and discontinuous symptom changes across the peripartum period and coded how the time point of the respective DASS-21 assessment at T1, T2, T3, T5, T6, and T7 was related to the time point of delivery. Table 1 provides more detailed information on how each predictor was coded.

**Table 1:** Description and coding of the examined predictors.

Predictor	Used to examine…	Coding
Linear age	Linear age effects	• Coded in years at T1, centered^a^ and divided by 10^b^
Quadratic age	Quadratic age effects	• Linear age variable^2^
Cubic age	Cubic age effects	• Linear age variable^3^
Linear symptom changes during pregnancy	Linear changes in depressive, anxiety, and stress symptoms from early until late pregnancy	• Coded with the time span (in months) between the respective assessment wave and the time point of delivery for all measurement occasions during pregnancy (T1, T2, and T3) (i.e., coded with -7.25 (-29/ 4) for T1 (week 10 to 12 of gestation), with -4.25 (-17/ 4) for T2 (week 22 to 24 of gestation), and with -1 (-4/ 4) for T3 (week 35 to 37 of gestation))^c^ • Coded with 0 for all measurement occasions after delivery (T5, T6, and T7)
Linear symptom changes after delivery	Linear changes in depressive, anxiety, and stress symptoms from two until 16 months postpartum	• Coded with the time span (in months) between the respective assessment wave and the time point of delivery for all measurement occasions after delivery (T5, T6, and T7) (i.e., coded with 2 for T5 (two months postpartum), with 4 for T6 (four months postpartum), and with 16 for T7 (16 months postpartum)) • Coded with 0 for all measurement occasions during pregnancy (T1, T2, and T3)
Short-term symptom changes after delivery	Abrupt and transient short-term changes in depressive, anxiety, and stress symptoms at two months postpartum (as compared to all other waves)	• Coded with 1 for T5 (two months postpartum) • Coded with 0 for all other measurement occasions (T1, T2, T3, T6, and T7)
Long-term symptom changes after delivery	Enduring long-term changes in depressive, anxiety, and stress symptoms more than two months after delivery (as compared to all previous waves)	• Coded with 1 for T6 (four months postpartum) and T7 (16 months postpartum) • Coded with 0 for all previous measurement occasions (T1, T2, T3, and T5)

^a^ The linear age variable was centered to ensure that the intercept in the overall model referred to an average-aged woman of the total sample

^b^ The linear age variable was divided by 10 to ensure that the quadratic and cubic age effects would not become too small to be reported rounded at three decimals

^c^ Because a few women ended their study participation during pregnancy we assumed an average length of pregnancy of 40 weeks and subtracted the average week of gestation, in which T1, T2, and T3 were conducted, from this length.

We built separate models per subscale (depression, anxiety, and stress) and modeled the effects as fixed effects. Because our statistical approach based on multilevel analyses enables to deal with missing data at individual waves our models refer to the total baseline sample (*N* = 306), including women who did or did not participate in subsequent waves.

In order to investigate whether peripartum depressive, anxiety, and stress symptoms varied as a function of maternal personality and perceived social support, we added the standardized score of the respective Big Five personality trait or perceived social support to the respective model. Because personality and perceived social support were assessed at T2 and 13 women were not retained from T1 until T2 the sample size in these analyses is slightly smaller (*N* = 293).

To examine the role of maternal personality and perceived social support for peripartum changes in depressive, anxiety, and stress symptoms, we added an interaction term between the respective event-related predictor and the standardized score of the respective Big Five personality trait or perceived social support to the respective model. To avoid mutlicollinearity, one interaction term was added at a time.

Finally, we repeated the analyses and included any lifetime depressive disorder (1 = ‘yes’ versus 0 = ‘no’) and any lifetime anxiety disorder (1 = ‘yes’ versus 0 = ‘no’) prior to pregnancy as additional predictors to control for lifetime diagnoses of depression and anxiety prior to pregnancy.

Because each analysis refers to an individual research question we did not adjust for multiple testing [85]. We set the alpha level at .01 (two-tailed testing).

## Results

### Peripartum changes in depressive, anxiety, and stress symptoms

Means and standard deviations for depressive, anxiety, and stress symptoms at T1, T2, T3, T5, T6, and T7 as well as averaged across all six waves are presented in Table 2. Peripartum changes in psychopathological symptoms are shown in Table 3. Significant linear change effects during pregnancy for depression (*b* = -0.055, *SE* = 0.010, *p* < .001) and for stress (*b* = -0.047, *SE* = 0.010, *p* < .001) indicated that depressive and stress symptoms gradually decreased from early until late pregnancy. Significant short-term change effects after delivery for anxiety (*b* = -0.193, *SE* = 0.069, *p* = .005) and for stress (*b* = 0.468, *SE* = 0.065, *p* < .001) indicated that anxiety symptoms were lower and stress symptoms were higher two months after delivery (T5) as compared to before. Similarly, significant long-term change effects after delivery for anxiety (*b* = -0.274, *SE* = 0.081, *p* = .001) and for stress (*b* = 0.320, *SE* = 0.076, *p* < .001) indicated that anxiety symptoms remained lower and stress symptoms remained higher four and 16 months after delivery (T6 and T7). All of these associations remained significant after adjustment for lifetime depressive and anxiety disorders prior to pregnancy.

**Table 2:** Means and standard deviations for depressive, anxiety, and stress symptoms at each wave and averaged across all six waves.

	T1		T2		T3		T5		T6		T7		Grand mean	
Number of observations	N = 306		N = 293		N = 278		N = 281		N = 283		N = 255		N = 1,696	
DASS-21	*M*	*SD*	*M*	*SD*	*M*	*SD*	*M*	*SD*	*M*	*SD*	*M*	*SD*	*M*	*SD*
Depression	4.65	4.60	3.15	3.44	3.25	3.62	3.15	3.96	3.13	4.10	3.19	4.36	3.44	4.07
Anxiety	2.38	3.43	1.92	3.14	2.42	3.41	1.60	3.05	1.31	3.39	1.14	2.76	1.81	3.25
Stress	11.15	6.85	9.26	5.99	9.20	6.33	11.53	6.56	10.46	6.72	9.77	6.98	10.25	6.63

DASS-21 = 21-item Depression Anxiety Stress Scale. *M* = Mean. *SD* = Standard Deviation. T1: week 10 to 12 of gestation; T2: week 22 to 24 of gestation; T3: week 35 to 37 of gestation; T4: 10 days postpartum; T5: two months postpartum; T6: four months postpartum; T7: 16 months postpartum

**Table 3:** Changes in depressive, anxiety, and stress symptoms across the peripartum period (N = 306).

	Depression			Anxiety			Stress		
Coefficient	*b*	*SE*	*p*	*b*	*SE*	*p*	*b*	*SE*	*p*
Intercept	-0.211	0.071	.003	0.091	0.070	.198	-0.235	0.072	.001
Linear age	0.068	0.165	.679	0.113	0.165	.495	0.309	0.178	.083
Quadratic age	0.280	0.187	.134	0.277	0.188	.140	-0.024	0.201	.906
Cubic age	-0.461	0.281	.100	-0.506	0.281	.072	-0.458	0.303	.130
Linear symptom changes during pregnancy	-0.055	0.010	< .001	0.001	0.010	.907	-0.047	0.010	< .001
Linear symptom changes after delivery	0.002	0.006	.738	-0.004	0.006	.488	-0.007	0.005	.167
Short-term symptom changes after delivery	0.099	0.070	.160	-0.193	0.069	.005	0.468	0.065	< .001
Long-term symptom changes after delivery	0.093	0.082	.257	-0.274	0.081	.001	0.320	0.076	< .001

*b*: Coefficient from multilevel mixed-effect models; *SE*: standard error; *p*: *p*-value; adjusted for linear, quadratic, and cubic age.

### The role of maternal personality

Correlations between the Big Five personality traits and perceived social support at T2 are presented in Table 4.

**Table 4:** Correlations between the Big Five personality traits and perceived social support at T2 (N = 293).

	Openness	Conscientiousness	Extraversion	Agreeableness	Emotional stability
	*r*	*r*	*r*	*r*	*r*
Openness	1.00				
Conscientiousness	0.18	1.00			
Extraversion	0.26	0.23	1.00		
Agreeableness	0.08	0.22	0.23	1.00	
Emotional stability	0.13	0.27	0.32	0.27	1.00
Perceived social support	0.09	0.18	0.36	0.30	0.30

Examining the role of maternal personality for peripartum depressive, anxiety, and stress symptoms (main effects) revealed that more conscientious women experienced lower depressive (*b* = -0.168, *SE* = 0.039, *p* < .001) and lower stress (*b* = -0.166, *SE* = 0.043, *p* < .001) symptoms across the peripartum period. The same was true for more extraverted women (depressive symptoms: *b* = -0.177, *SE* = 0.039, *p* < .001; stress symptoms: *b* = -0.147, *SE* = 0.043, *p* = .001). Most notably, more emotionally stable women experienced lower depressive (*b* = -0.328, *SE* = 0.035, *p* < .001), lower anxiety (*b* = -0.294, *SE* = 0.037, *p* < .001), and lower stress (*b* = -0.415, *SE* = 0.035, *p* < .001) symptoms across the peripartum period. All of these associations remained significant after adjustment for lifetime depressive and anxiety disorders prior to pregnancy.

Examining the role of maternal personality for peripartum changes in depressive, anxiety, and stress symptoms revealed the following interactive effects: First, emotional stability interacted with the linear change effect during pregnancy on anxiety symptoms (*b* = 0.019, *SE* = 0.007, *p* = .005). That is, more emotionally stable women more strongly increased in anxiety symptoms from early until late pregnancy. Second, extraversion interacted with the linear change effect after delivery on depression (*b* = -0.010, *SE* = 0.003, *p* = .003). That is, more extraverted women less strongly increased in depressive symptoms from two to 16 months postpartum. Both interactive effects remained significant after adjustment for lifetime depressive and anxiety disorders prior to pregnancy.

### The role of perceived social support

Investigating the role of perceived social support revealed that women with higher perceived social support experienced lower depressive (*b* = -0.308, *SE* = 0.036, *p* < .001), lower anxiety (*b* = -0.225, *SE* = 0.039, *p* < .001), and lower stress (*b* = -0.304, *SE* = 0.040, *p* < .001) symptoms across the peripartum period (main effects). All of these associations remained significant after adjustment for lifetime depressive and anxiety disorders prior to pregnancy. However, there was no evidence that perceived social support interacted with the event-related predictors on depressive, anxiety, or stress symptoms (all *p*-values>.01), indicating that peripartum symptom changes did not vary by perceived social support.

## Discussion

Becoming a mother constitutes a significant turning point in life. During pregnancy and after delivery, (expectant) mothers are confronted with manifold changes concerning their body, their family, and their social identity [1–4]. As suggested by previous research, their personality [54–63] and levels of perceived social support [5, 69–71] might crucially affect how they adjust to these changes and whether they do or do not experience alterations in depressive, anxiety, and stress symptoms across the peripartum period. Psychopathological symptoms and elevated distress during pregnancy and after delivery have been associated with multiple adversities (e.g., an impaired mother-child-relationship) [13–21], which in turn have been linked to developmental problems in the offspring [12, 18–21]. Therefore, examining the predictive role of specific personality traits and perceived social support for peripartum changes in psychopathological symptoms is crucial to improve an early recognition of high-risk women, who might profit from targeted prevention [22, 23].

In this study, we used prospective-longitudinal data from a regional-epidemiological community sample of 306 expectant mothers to examine the role of the Big Five personality traits and perceived social support in pregnancy for changes in depressive, anxiety, and stress symptoms from early pregnancy until 16 months postpartum. Our main findings were as follows: Depressive and stress symptoms decreased from early to late pregnancy. Anxiety symptoms were lower, whereas stress symptoms were higher after delivery as compared to pregnancy. Across the peripartum period, depressive and stress symptoms were lower in more conscientious and more extraverted women, and depressive, anxiety, and stress symptoms were lower in more emotionally stable women. All of the examined psychopathological symptoms were lower in women with higher perceived social support. Moreover, more emotionally stable women more strongly increased in anxiety symptoms during pregnancy, and more extraverted women less strongly increased in depressive symptoms after delivery. The results remained stable when considering lifetime diagnoses of depressive and anxiety disorders prior to pregnancy.

### Peripartum changes in depressive, anxiety, and stress symptoms

Our findings that depressive and stress symptoms decreased during pregnancy are consistent with previous evidence that psychopathological symptoms remained stable or improved across the peripartum period [24–31]. These results might be explained by a range of ‘buffering’ pregnancy-related physiological changes, which have been associated with alterations in mood and psychophysiological stress responsivity [32–36]. The fact that anxiety symptoms were lower after delivery might be due to the possibility that many expectant mothers experienced pregnancy- and childbirth-related anxieties, which diminished after their child was born. After delivery, new mothers had to adjust to their novel role as a mother and associated socio-environmental changes [86]. Many of them might have felt initially overwhelmed and experienced a lack of sleep [87] due to childcare as well as possibly increased conflicts and distress with their partner [1, 88], which might explain higher stress symptoms after delivery.

### The role of maternal personality

Examining the role of personality revealed that more emotionally stable women experienced lower depressive, anxiety, and stress symptoms across the peripartum period. These results correspond to cross-sectional and longitudinal findings that more emotionally stable women were at lower risk of peripartum psychopathological symptoms and mental disorders [54–60].

At the same time, we found that more emotionally stable women more strongly increased in anxiety symptoms during pregnancy. One might speculate whether more emotionally stable women were less familiar with feelings of fear and anxiety prior to pregnancy. Due to ‘normal’ pregnancy- and childbirth-related fears and anxieties [15] they might have perceived a higher subjective increase of anxiety during pregnancy (because they had rarely dealt with fear and anxiety before). However, additional research is needed to test this assumption along with other possible mechanisms that might explain this result.

Consistent with some previous research [55, 61–63], we further found that more conscientious and more extraverted women experienced fewer depressive and stress symptoms across the peripartum period. Possibly, more conscientious women were characterized by a higher sense of mastery and more effective self-regulation strategies [64, 68] and thus managed to adjust to their novel role as a (an expectant) mother more easily. More extraverted women might have more actively engaged in social activities before, during, and after their pregnancy and thus had a denser social network and higher social support [67]. Based on previous research, it is further plausible to assume that more extraverted women tended to experience more positive affect in social and non-social situations [64–66], which might explain why they increased less strongly in depressive symptoms after delivery.

### The role of perceived social support

Examining the role of perceived social support revealed that women with higher perceived social support in pregnancy experienced fewer depressive, anxiety, and stress symptoms across the peripartum period. These findings are consistent with previous evidence linking higher perceived social support to a lower risk of peripartum psychopathological symptoms and mental disorders [5, 89]. Women with higher informational, instrumental, and emotional support might have managed to prepare for and adjust to their novel role as a mother more easily. In addition, these women might have felt more accepted and secure, leading to a lower risk of depression and anxiety.

However, we did not find that peripartum changes in depressive, anxiety, or stress symptoms varied by perceived social support in pregnancy, which is inconsistent with recent evidence from Racine et al. [71]. Possibly, these inconsistencies are due to a range of methodological differences concerning the study sample, study design, assessment instruments, and statistical analyses. For example, Racine et al. [71] examined cross-lagged associations of perceived social support with anxiety and stress symptoms across the peripartum period. They found that within-person increases in partner or family support were associated with symptom decreases at a later point of time. In contrast, our study investigated whether women with varying levels of perceived social support in pregnancy differed with respect to continuous and discontinuous short- and long-term changes in depressive, anxiety, and stress symptoms from early pregnancy until late postpartum. The fact that no such differences were found suggests that peripartum symptom changes were comparable between women with low and high support levels in pregnancy. Although women who felt more supported experienced fewer symptoms, perceived social support appeared to be less predictive of symptom changes as compared to other factors (e.g., maternal personality as well as depressive and anxiety disorders prior to pregnancy [31]). Besides, our sample was characterized by relatively high average social support levels, which might also explain this result.

### Strengths and limitations

Our study has several strengths: The Big Five personality traits and perceived social support were assessed in pregnancy, and depressive, anxiety, and stress symptoms were assessed prospectively at three times during pregnancy and three times after delivery with well-established questionnaires. Based on these data, we examined continuous and discontinuous symptom changes from early pregnancy until 16 months postpartum and tested whether these changes varied by maternal personality and perceived social support. Additionally, we controlled for depressive and anxiety disorders prior to pregnancy.

Nonetheless, our study is not without limitations: First, the DASS-21 is a relatively short instrument (seven items per symptom subscale) and primarily refers to internalizing symptoms. Therefore, additional research is needed to investigate the role of maternal personality and perceived social support for peripartum changes in other (e.g., externalizing) symptoms.

Second, although or study was based on a prospective-longitudinal design, maternal personality and perceived social support were assessed at T2 for the first time. For this reason, we cannot rule of the possibility that maternal personality and perceived social support already changed from T1 and T2 and that these changes were affected by previous change in depressive, anxiety, and stress symptoms. However, personality traits are defined to be relatively consistent across different situations and over time [90–93].

Third, 89.5% of the initial study sample was retained from T1 until T7 and all women who participated at individual waves (except for three women at T5 and twelve women at T7) provided information on their personality (T2), perceived social support (T2), and psychopathological symptoms (T1, T2, T3, T5, T6, and T7). Although this retention rate is relatively high [6, 72], systematic drop out might have occurred.

Fourth, additional research is needed to study the underlying mechanisms and associated adverse outcomes of specific unfavorable peripartum changes in psychopathological symptoms (e.g., altered DNA methylation [10, 11]) in (expectant) mothers and their infants.

Fifth, our findings stem from a regional-epidemiological sample from Dresden and might not be generalizable to pregnant women in general [6, 72].

### Conclusions

Our findings suggest that especially less emotionally stable, less conscientious, and less extraverted women as well as women with lower perceived social support are at increased risk for elevated psychopathological symptoms throughout the peripartum period and might thus profit from targeted prevention and early intervention [22, 23].

## Supporting information

S1 File (DOCX)
