# Supplementary material for: Maternal personality, social support, and changes in depressive, anxiety, and stress symptoms during pregnancy and after delivery: A prospective-longitudinal study
Source: PLoS One. 2020 Aug 24;15(8):e0237609. doi: 10.1371/journal.pone.0237609 (PMC7446870; doi:10.1371/journal.pone.0237609)
Supplement: S1 File — (DOCX) [file pone.0237609.s001.docx]

**Financial disclosure statement**

This work has been supported by the Institute of Clinical Psychology and Psychotherapy, Technische Universität Dresden and by the Lundbeck Institute Skodsborg, Denmark. Parts of the field work were additionally funded by the Friends and Sponsors (Gesellschaft der Freunde und Förderer) of the Technische Universität Dresden.

**Competing interests**

Prof. Dr. Hans-Ulrich Wittchen reports the following items that might be perceived as a potential conflict of interest: Prof. Dr. Hans-Ulrich Wittchen is on the advisory board of the Lundbeck Institute Skodsborg, Denmark and has also received grant support from there. This does not alter our adherence to PLOS ONE policies on sharing data and materials. All other authors declare that they have no financial relationships that might be perceived as a potential conflict of interest.

**Ethical standards**

The authors assert that all procedures contributing to this work comply with the ethical standards of the relevant national and institutional committees on human experimentation and with the Helsinki Declaration of 1975, as revised in 2013.

**Contributors**

Eva Asselmann and Julia Martini contributed to the conception of the current work. Eva Asselmann conducted the analyses and wrote the manuscript draft. Stefanie Kunas and Julia Martini provided critical revision and contributed to the interpretation of findings.

**Data availability statement**

Due to the sensitive nature of the questions asked in this study and the personal observations of mothers and their infants, participants were assured that all raw data will remain confidential and will not be shared. The Ethics Committee of the Medical Faculty of the Technische Universität Dresden did not approve that openly accessible data can be shared in spite of these serious restrictions.
